# Identification of Nuclear and Cytoplasmic mRNA Targets for the Shuttling Protein SF2/ASF

**DOI:** 10.1371/journal.pone.0003369

**Published:** 2008-10-08

**Authors:** Jeremy R. Sanford, Pedro Coutinho, Jamie A. Hackett, Xin Wang, William Ranahan, Javier F. Caceres

**Affiliations:** 1 MRC Human Genetics Unit, Western General Hospital, Edinburgh, United Kingdom; 2 Department of Biochemistry and Molecular Biology, Indiana University School of Medicine, Indianapolis, Indiana, United States of America; Lehigh University, United States of America

## Abstract

The serine and arginine-rich protein family (SR proteins) are highly conserved regulators of pre-mRNA splicing. SF2/ASF, a prototype member of the SR protein family, is a multifunctional RNA binding protein with roles in pre-mRNA splicing, mRNA export and mRNA translation. These observations suggest the intriguing hypothesis that SF2/ASF may couple splicing and translation of specific mRNA targets in vivo. Unfortunately the paucity of endogenous mRNA targets for SF2/ASF has hindered testing of this hypothesis. Here, we identify endogenous mRNAs directly cross-linked to SF2/ASF in different sub-cellular compartments. Cross-Linking Immunoprecipitation (CLIP) captures the *in situ* specificity of protein-RNA interaction and allows for the simultaneous identification of endogenous RNA targets as well as the locations of binding sites within the RNA transcript. Using the CLIP method we identified 326 binding sites for SF2/ASF in RNA transcripts from 180 protein coding genes. A purine-rich consensus motif was identified in binding sites located within exon sequences but not introns. Furthermore, 72 binding sites were occupied by SF2/ASF in different sub-cellular fractions suggesting that these binding sites may influence the splicing or translational control of endogenous mRNA targets. We demonstrate that ectopic expression of SF2/ASF regulates the splicing and polysome association of transcripts derived from the SFRS1, PABC1, NETO2 and ENSA genes. Taken together the data presented here indicate that SF2/ASF has the capacity to co-regulate the nuclear and cytoplasmic processing of specific mRNAs and provide further evidence that the nuclear history of an mRNA may influence its cytoplasmic fate.

## Introduction

Eukaryotic messenger RNA (mRNA) must be processed prior to programming protein synthesis. The minimal modifications for most mRNAs include capping, pre-mRNA splicing and polyadenylation [1]. These reactions occur in the nucleus and must be completed prior to nuclear export of the mRNA to the cytoplasm. The cytoplasmic fate of mRNA is also subject to regulation at the level of localization, stability and translational efficiency [2]. RNA processing reactions have been extensively studied using biochemical systems; however, these are functionally linked in living cells providing increased efficiency and regulatory potential for gene expression [3]. The molecular mechanisms responsible for the coupling of post-transcriptional regulatory networks are poorly understood. A subset of multi-functional mRNA binding proteins operating at the interface of distinct RNA processing machineries may contribute to coupling of post-transcriptional gene expression.

The Serine and Arginine-rich (SR) protein family consists of eight phylogenetically conserved proteins ranging in molecular weight from 20–75 kDa. SR proteins have well-characterized roles in pre-mRNA splicing including exon definition and assembly of the mature spliceosome [4], [5]. A subset of the SR protein family also function outside pre-mRNA splicing [6]. Indeed, we recently established a role of the shuttling SR protein SF2/ASF in mRNA translation [7], [8]. SF2/ASF enhances translation initiation by promoting phosphorylation of the translational repressor protein 4E-BP1 by the mammalian target of rapamycin (mTOR) [9]. Further roles for SRp20 and 9G8 in internal ribosome entry site (IRES)-mediated translation and intronless mRNA export have been documented [10], [11]. These observations suggest the hypothesis that shuttling SR proteins may couple nuclear and cytoplasmic steps of pre-mRNA processing. Elucidating endogenous mRNA targets that are regulated by SF2/ASF in both the nucleus and cytoplasm is critical to testing the validity of this hypothesis.

The cross-linking immunoprecipitation (CLIP) method is a powerful method for elucidating the target specificity of RNA binding proteins *in vivo* [12]. In this protocol, living cells are exposed to UV irradiation to induce covalent cross-links between RNA binding proteins and their *in situ* RNA targets. Prior to immunoprecipitation of a specific RNA binding protein lysates are treated with RNase in order to generate 40–60 nt cross-linked RNA tags. Co-purifying RNA tags are then cloned and sequenced revealing genomic locus of the RNA as well as the position of the RNA-protein interaction within the RNA molecule. CLIP analysis of the neural specific splicing factor Nova identified a post-transcriptional regulatory network related to synaptic function. Mechanistic studies determined the relationship between positions of Nova binding sites and effects on splice site selection [13], [14]. These data were used to build an RNA map capable of predicting Nova-dependent splice site selection [15]. Here, we use CLIP to identify RNA targets for the shuttling SR protein, SF2/ASF, in order to test the hypothesis that this SR family protein member regulates nuclear and cytoplasmic expression of specific mRNAs in living cells. This sampling of nuclear, cytoplasmic and polyribosome-associated mRNA targets suggests that SF2/ASF may remain associated with a subset of specific transcripts as they are trafficked from the nucleus to the cytoplasm. Furthermore we provide evidence that SF2/ASF couples alternative splicing with enhanced translation for several endogenous mRNAs. These data provide insights to the targets and roles of an essential shuttling RNA binding protein in coupling nuclear and cytoplasmic steps of post-transcriptional gene expression.

## Results

### Cross-linking immunoprecipitation of SF2/ASF

**Figure 1:**
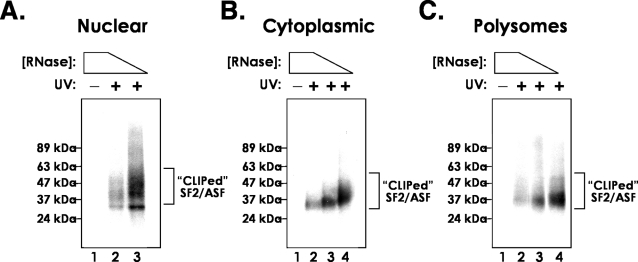
Cross-linking immunoprecipitation of SF2/ASF from different HEK293T cellular fractions. SF2/ASF-RNA complexes, indicated by the bracket, were visualized by autoradiography. Positions of molecular weight standards are given at the left of each panel and exposure of cells to ultra violet radiation (UV) is indicated by + or −.

Previously, we have shown that SF2/ASF binds directly to cytoplasmic mRNA *in vivo* and enhances the translation of reporter mRNAs both *in vitro* and *in vivo* [8], suggesting that SF2/ASF may regulate the nuclear and cytoplasmic fate of specific endogenous mRNAs. Despite nearly two decades of study, few endogenous targets of SR proteins have been identified. In order to determine if SF2/ASF can regulate nuclear and cytoplasmic processing of endogenous mRNAs we used the CLIP protocol to identify binding sites for SF2/ASF in mRNAs from different subcellular fractions. Nuclear, cytoplasmic and polyribosome-containing sub-cellular fractions were prepared as previously described [7] from control cells or those exposed to UV irradiation and cell extracts were treated with decreasing amounts of RNase A/T1 to partially degrade cross-linked RNA molecules. SF2/ASF was immunoprecipitated from each extract under stringent conditions. Purified SF2/ASF-RNA complexes were modified on beads by the addition of a 3′ RNA linker and ^32^P at the 5′ end. Complexes were resolved on 10% Novex Nupage gels (Invitrogen, USA) and transferred to nitrocellulose. Fig. 1 shows UV-dependent, RNase-sensitive complexes immunoprecipitated by the anti-SF2/ASF monoclonal antibody. Note the increased molecular weight of the complex as RNase concentrations are decreased. The heterogeneous migration of these complexes is due to increasing length of captured RNA fragments. Complexes between 37 and 42 kDa were purified from nitrocellulose membranes. RNA was extracted, ligated to a 5′ RNA linker and amplified by RT-PCR (not shown). Amplicons were then concatamerized, ligated in pcDNA3.1, cloned and sequenced.

### Mapping of SF2/ASF mRNA targets

**Figure 2:**
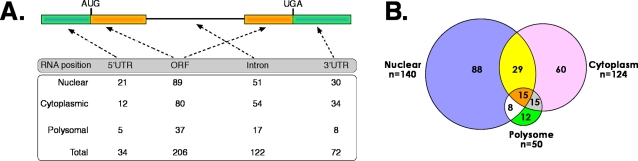
Distribution of SF2/ASF binding sites in relation to gene structure and sub-cellular localization. A) The “coding” SF2/ASF CLIP tags from nuclear, cytoplasmic and polysomal sets were analyzed in relation to their relative position within genes. This showed that the majority map to intronic or to coding exonic sequences and only a small proportion mapped to UTRs. B) Venn diagram of exonic SF2/ASF binding sites from Nuclear, cytoplasmic and polysomal sub-cellular fractions. The number of unique binding sites identified in each fraction is designated as “n”.

We obtained sequences from more than 3,500 amplicons corresponding to putative SF2/ASF RNA targets. We used BLAT to align sequences to the human genome allowing for two mis-matches or gaps within the alignment [16]. For this analysis we focused on RNA fragments corresponding to protein coding genes, any other sites were ignored. 1,237 amplicons could be mapped to a total of 326 positions in 180 protein coding genes; 43% of the binding sites were represented by multiple sequences. A majority of binding sites (68%) fell within exonic sequences (Fig. 2A). We also detected binding sites for SF2/ASF within intronic sequences. However, as shown below, we were unable to detect a consensus motif within the pool of intronic binding sites, therefore we focused the remaining analysis on the exonic pool of sequences. We compared the binding specificity of SF2/ASF RNA targets identified in different sub-cellular compartments in order to determine if any RNA transcripts were in common. We found that 72 exonic binding sites are engaged by SF2/ASF in both the nucleus and the cytoplasm or polysome fraction. Interestingly, 15 binding sites in 12 different genes are engaged by SF2/ASF in all three compartments (Fig. 2B). These data provide strong evidence that SF2/ASF associates with a subset of messenger RNAs in the nucleus and that the same *cis*-acting element is recognized in the cytoplasm. We hypothesize that SF2/ASF remains bound to the mRNA as the mRNA is trafficked from the nucleus to the cytoplasm [7].

### Identification of a consensus binding site for SF2/ASF

**Figure 3:**
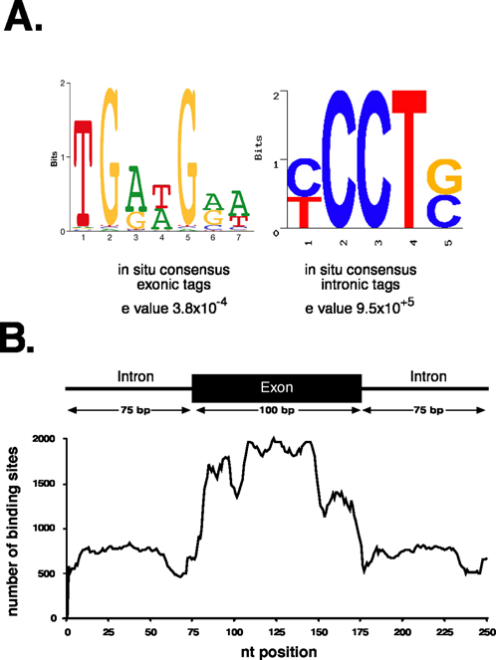
Identification of the SF2/ASF consensus binding site and its frequency distribution in coding exons and flanking introns. A) The exonic or intronic clip tags were analyzed using MEME to identify sequence patterns that are over-represented. MEME was unable to identify a consensus motif within the intronic binding sites. B) This pattern was used to analyze composite sequences made for all exons with more than 100 nucleotides, from protein coding genes. This analysis shows that the pattern is over-represented within exonic sequences and in particular within the exon 5′ prime and middle of the exons.

The *in vitro* RNA binding specificity of many SR proteins including SF2/ASF, have been well-characterized using approaches such as selected evolution of ligands by exponential enrichment (SELEX) [17]. Different consensus sites for SF2/ASF have been obtained using different SELEX strategies [18], [19]. We used the motif finding algorithm MEME, to search exonic or intronic RNA fragments for over represented sequences [20], [21]. Fig. 3A shows that a purine-rich consensus motif is present within exonic sequences targeted by SF2/ASF with a highly significant expectation value (e value) of 3.8×10^−4^. No other statistically significant motifs were identified within the exonic sequences. By contrast MEME was unable to detect an enriched sequence motif within the intronic pool of binding sites identified by CLIP. These data suggest that intronic binding sites may prove to be a nonspecific contaminant. To test the validity of the consensus model, we asked if the consensus sequence is enriched in exonic or intronic sequences across the genome using composite exons [18]. We then used the position weight matrix calculated from the consensus motif to determine the number of binding sites at each position across all intron/exon/intron composite sequences. Fig. 3B demonstrates that the consensus motif for SF2/ASF is highly enriched at the edges of exons relative to intronic sequences or the interior of exon sequences. These data support the hypothesis that SF2/ASF plays a role in the establishment of exon identity.

### Annotation of alternatively spliced pre-mRNA targets of SF2/ASF

SR proteins have well characterized roles in both constitutive and alternative pre-mRNA splicing. Resolving these dual functions of SR proteins solely by the use of heterologous substrates and mini-gene reporters has proven difficult. In order to determine if the endogenous binding sites for SF2/ASF may reveal any clues to the roles of SR proteins in alternative splicing we manually curated annotated ENSEMBL transcripts targeted by SF2/ASF for examples of alternative splicing using two distinct databases: AceView and FAST-DB [22], [23]. Of the 234 exonic binding sites identified by CLIP, only 72 were subject to some form of alternative processing (Table 1–4 for details on binding sites associated with specific types of alternative splicing). We also identified 9 binding sites within constitutive exons located downstream and adjacent to alternative cassette exons (See Table 5). This type of arrangement suggests that SF2/ASF may promote skipping of the upstream cassette exon, as recently demonstrated for alternative splicing of the receptor tyrosine kinase RON during breast cancer metastasis [24]. Within this list of alternative exons targeted by SF2/ASF there is a modest enrichment for genes encoding RNA binding proteins (SFRS1, PABPC1, hnRNPDL, hnRNPAB, SERBP1, RPL5, SON, RPL0, RPS10, RPL12, RPS24, RPL19, PKRA) and genes involved in regulation of biological processes such as cell division, proliferation and apoptosis (CDK4, MAPK3, ACIN1). The ENSA gene is also of interest as it is bound by SF2/ASF in each sub-cellular compartment and is a Type 2 diabetes candidate gene. ENSA is an endogenous ligand for the sulfonylurea receptor which plays an important role in insulin release from pancreatic beta cells [25], [26]. Several of the alternative splicing events involving exons targeted by SF2/ASF lead to premature termination codons and potentially NMD (SFRS1, CDK4, MAPK3, for example) however the majority of alternative events alter the primary structure of the encoded polypeptide (not shown). All of these splicing events can be visualized using the Friendly Alternative Splicing and Transcript Diversity database (http://www.fast-db.com; [22]).

**Table 1:** Annotation of alternative cassette exons bound by SF2/ASF.

*Partial list of alternative cassette exons bound by SF2/ASF*					
Gene ID	Exon Position	Gene Description	Localization	Gene Family	Function
ACIN1	12	apoptotic chromatin condensation inducer 1	Nucleus	enzyme	apoptotic chromosome condensation
BECN1	11	beclin 1 (coiled-coil, myosin-like BCL2 interacting protein)	Nucleus; Polysome	other	anti-apoptosis;cellular defense response
CALM2	4	calmodulin 2 (phosphorylase kinase, delta)	Cytoplasm	other	Calcium Binding;G-protein coupled receptor protein signaling pathway
DHX15	5	DEAH (Asp-Glu-Ala-His) box polypeptide 15	Cytoplasm	enzyme	mRNA processing
ERCC6	2	excision repair cross-complementing rodent repair deficiency, complementation group 6	Cytoplasm	transcription regulator	transcription-coupled nucleotide-excision repair
MAPK3	6	mitogen-activated protein kinase 3	Cytoplasm	kinase	intracellular signaling cascade
NUP43	4	nucleoporin 43kDa	Nucleus; Cytoplasm; Polysome	transporter	nuclear envelope
PIP5K2A	7	phosphatidylinositol-5-phosphate 4-kinase, type II, alpha	Nucleus	kinase	glycerophospholipid metabolic process
SON	3	SON DNA binding protein	Nucleus	other	anti-apoptosis
TCF12	9	transcription factor 12 (HTF4, helix-loop-helix transcription factors 4)	Cytoplasm	transcription regulator	regulation of transcription from RNA polymerase II promoter
WDR42A	11	WD repeat domain 42A	Nucleus; Cytoplasm	other	Unknown
Y13871	1	hypothetical protein LOC202181	Nucleus; Cytoplasm; Polysome	other	Unknown
ZHX1	3	zinc fingers and homeoboxes 1	Nucleus	transcription regulator	negative regulation of transcription, DNA-dependent

Exons were annotated manually according to AceView and FAST-DB databases. Column Headers: Gene ID, official gene symbol; Exon position, number of exon within the primary transcript; Gene Description; Localization, the sub-cellular fraction used for CLIP experiment; Gene Family; Function, the primary encoded function of gene. This table is a partial list of cassette exons bound by SF2/ASF. The complete list can be found in Table S1.

**Table 2 pone-0003369-t002:** Annotated as described in Table 1.

*SF2/ASF target exons containing alternative 3′ splice sites*					
Gene ID	Exon Position	Gene Description	Localization	Gene Family	Function
PSAP	7	prosaposin	Nucleus	other	glycosphingolipid metabolic process; lipid transport
HN1	3	hematological and neurological expressed 1	Nucleus	other	Not Determined
IGF1R	21	insulin-like growth factor 1 receptor	Nucleus	transmembrane receptor	insulin receptor signaling pathway
PRELID1	5	PRELI domain containing 1	Nucleus	other	multicellular organismal development
CALM2	3	calmodulin 2 (phosphorylase kinase, delta)	Cytoplasm	other	Calcium Binding; G-protein coupled receptor protein signaling pathway
AHCY	10	S-adenosylhomocysteine hydrolase	Cytoplasm	enzyme	one-carbon compound metabolic process
ZFP91	11	zinc finger protein 91 homolog (mouse)	Cytoplasm	transcription regulator	
UBE3B	28	ubiquitin protein ligase E3B	Cytoplasm	enzyme	Protein degredation
RPL10A	6	ribosomal protein L10a	Cytoplasm	other	Ribosome Component
GK	17	glycerol kinase	Cytoplasm	kinase	
RPS10	3	ribosomal protein S10	Nucleus; Cytoplasm	other	Ribosome Component
MGST3	2	microsomal glutathione S-transferase 3	Nucleus; Cytoplasm	enzyme	lipid metabolic process; signal transduction
RPL19	6	ribosomal protein L19	Nucleus; Cytoplasm	other	Ribosome Component
NAGPA	5	N-acetylglucosamine-1-phosphodiester alpha-N-acetylglucos-aminidase	Nucleus; Cytoplasm	enzyme	carbohydrate metabolic process; lysosome organization and biogenesis
B2M	2	beta-2-microglobulin	Nucleus; Cytoplasm	transmembrane receptor	immune response
ETNK1	2	ethanolamine kinase 1	Cytoplasm; Polysome	kinase	Phosphatidylethanol-amine biosynthetic process
EEF1A1	3,6	eukaryotic translation elongation factor 1 alpha 1	Nucleus; Cytoplasm; Polysome	translation	translational elongation
FLJ00313	7	Not Determined	Nucleus	Unknown	Unknown
NR2C1	12	nuclear receptor subfamily 2, group C, member 1	Nucleus	transcription regulator	Unknown
PWP1	13	PWP1 homolog (S. cerevisiae)	Nucleus	other	Unknown
CENPO	5	centromere protein O	Nucleus	other	role in cell growth and/or transcription
TCF12	9	transcription factor 12	Cytoplasm	transcription regulator	Regulation of transcription by RNA Polymerase II

**Table 3 pone-0003369-t003:** Annotated as described in Table 1.

*SF2/ASF target exons containing alternative 5′ splice sites*					
Gene ID	Exon Position	Gene Description	Localization	Gene Family	Function
NAGPA	5	N-acetylglucosamine-1-phosphodiester alpha-N-acetylglucosaminidase	Nucleus; Cytoplasm	enzyme	carbohydrate metabolic process; lysosome organization and biogenesis
SERBP1	6	SERPINE1 mRNA binding protein 1	Nucleus	other	mRNA processing
CDK4	2	cyclin-dependent kinase 4	Nucleus	kinase	Cell Cycle Regulation
CDC14A	15	CDC14 cell division cycle 14 homolog A (S. cerevisiae)	Nucleus	phosphatase	dual specificity protein tyrosine phosphatase
BCOR	4	BCL6 co-repressor	Cytoplasm	transcription regulator	Transcriptional corepressor
RPL5	1	ribosomal protein L5	Cytoplasm	other	Ribosome Component
NOL1	16	nucleolar protein 1, 120kDa	Polysome	other	Cell Cycle Regulation; RNA Methyltransferase
PKM2	2	pyruvate kinase, muscle	Cytoplasm; Polysome	kinase	Glycolytic enzyme
ETNK1	2	ethanolamine kinase 1	Cytoplasm; Polysome	kinase	phosphatidylethanolamine biosynthetic process
MAPK3	6	mitogen-activated protein kinase 3	Cytoplasm	kinase	intracellular signaling cascade
EEF1A1	2,3,6	eukaryotic translation elongation factor 1 alpha 1	Nucleus; Cytoplasm; Polysome	translation	translational elongation

**Table 4:** Annotated as described in Table 1.

*SF2/ASF target exons containing a retained intron*					
Gene ID	Exon Position	Gene Description	Localization	Gene Family	Function
ATP5B	2	ATP synthase, H+ transporting, mitochondrial F1 complex, beta polypeptide	Nucleus; Cytoplasm	transporter	generation of precursor metabolites and energy
ATP7B	2	ATPase, Cu++ transporting, beta polypeptide	Nucleus	transporter	cellular copper ion homeostasis
EEF1A1	1	eukaryotic translation elongation factor 1 alpha 1	Nucleus; Cytoplasm; Polysome	translation regulator	translational elongation
GNL3	13	guanine nucleotide binding protein-like 3 (nucleolar)	Cytoplasm	other	Stem Cell Proliferation
NAGPA	5	N-acetylglucosamine-1-phosphodiester alpha-N-acetylglucosaminidase	Nucleus; Cytoplasm	enzyme	carbohydrate metabolic process; lysosome organization and biogenesis
RAN	7	RAN, member RAS oncogene family	Nucleus	enzyme	GTP-binding protein involved in nucleocytoplasmic transport
RPL12	7	ribosomal protein L12	Nucleus	other	Ribosome Component
RPL23	3	ribosomal protein L23	Polysome	other	Ribosome Component
RPLP0	5	ribosomal protein, large, P0	Nucleus	other	Ribosome Component
SFRS1	4	splicing factor, arginine/serine-rich 1 (splicing factor 2, alternate splicing factor)	Cytoplasm	other	mRNA processing
TH1L	15	TH1-like (Drosophila)	Cytoplasm	other	Transcription regulator
TSSC4	4	tumor suppressing subtransferable candidate 4	Nucleus	other	Unknown
WDR59	12	WD repeat domain 59	Nucleus	transporter	Not Determined
ZMYM3	7	zinc finger, MYM-type 3	Cytoplasm	other	multicellular organismal development

**Table 5:** Annotated as described in Table 1.

*SF2/ASF target exons located adjacent to alternative cassette exons*					
Gene ID	Exon Position	Gene Description	Localization	Gene Family	Function
NETO2	9	neuropilin (NRP) and tolloid (TLL)-like 2	Cytoplasm	Transmembrane Protein	development; neuronal function
PRKRA	4	protein kinase, interferon-inducible double stranded RNA dependent activator	Cytoplasm	Kinase	Pro-apoptosis
RPS24	6	ribosomal protein S24	Cytoplasm	Ribosome Component	Translation factor
PABPC1	12	poly(A) binding protein, cytoplasmic 1	Nucleus; Polysome	Other	translation regulator
BRD2	13	bromodomain containing 2	Nucleus	kinase	generation of spermatozoa
YWHAH	2	tyrosine 3-monooxygenase/tryptophan 5-monooxygenase activation protein, eta polypeptide	Nucleus; Cytoplasm	Other	transcription regulator
HNRPDL	9	heterogeneous nuclear ribonucleoprotein D-like	Nucleus	Other	mRNA processing
HNRPAB	8	heterogeneous nuclear ribonucleoprotein A/B	Nucleus	Other	mRNA processing
MPZL1	6	myelin protein zero-like 1	Nucleus; Cytoplasm	Transmembrane Protein	Receptor

### Regulation of endogenous nuclear and cytoplasmic mRNA processing by SF2/ASF

**Figure 4:**
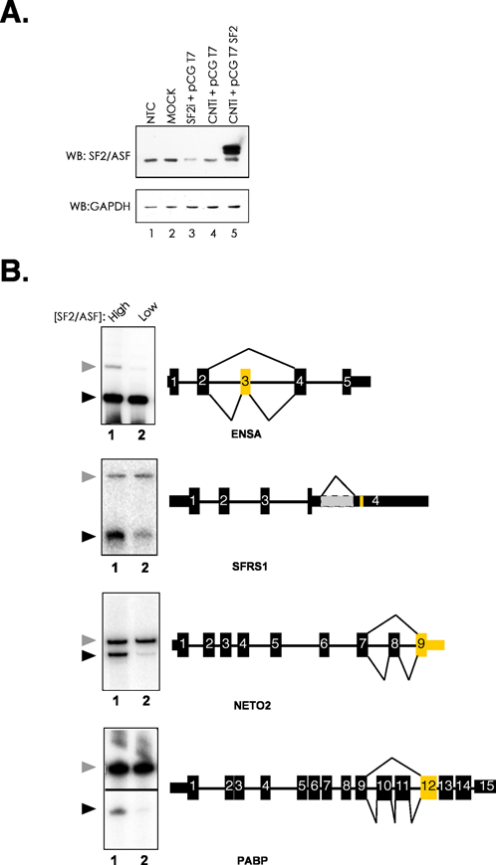
Alternative splicing of endogenous RNA transcripts targeted by SF2/ASF. A) Western blot analysis of HEK293T cells co-transfected with siRNA duplexes and expression plasmids. The upper panel is probed with anti-SF2/ASF monoclonal antibody, the lower panel is probed with anti-GAPDH monoclonal antibody. Lane 1, non transfected control (NTC); Lane 2, Mock (no siRNA or plasmid); Lane 3, siRNA targeting SF2/ASF and empty vector (pCGT7); Lane 4, scrambled siRNA and empty vector; Lane 5, scrambled siRNA and T7-tagged SF2/ASF expression plasmid (pCGT7 SF2). Total RNA was purified for RT-PCR analysis from samples corresponding to lanes 3 and 5. B) RT-PCR analysis of alternative splicing. Radiolabeled products were visualized by phosphorimaging. Schematic diagrams show exon-intron structure of target genes and patterns of alternative splicing. Thin rectangles indicate untranslated regions, thick rectangles indicate coding regions, thick lines indicate introns and thin lines indicate the alternative splicing event. Exons identified by CLIP are yellow. The grey region in the SFRS1 3′UTR indicates a retained intron. Arrowheads to the left of each panel indicate the included (gray arrowhead) and skipped (black arrowhead) alternative mRNA isoform. Lane 1 and 2 contain RNA purified from SF2/ASF overexpressing and depleted cells, for example see panel A, lanes 5 and 3, respectively. The sub-cellular fraction corresponding to the initial CLIP experiment is indicated below each gene symbol.

A majority of RNA fragments that co-purified with SF2/ASF contain a purine-rich consensus motif, reminiscent of previous SELEX binding sites [19]. Interestingly, a subset of mRNA targets was bound by SF2/ASF in both the nucleus and the cytoplasm. Three of these targets, ENSA (endosulfine α), PABPC1 (poly A binding protein 1) and NETO2 (neuropillin-like tolloid receptor 2) contained binding sites for SF2/ASF within or flanking alternative cassette exons, whereas the SFRS1 (pre-mRNA encoding SF2/ASF) contains a binding site adjacent to a retained intron. We then asked whether SF2/ASF modulates the alternative splicing of these pre-mRNAs in the nucleus and enhance their translation in the cytoplasm. We manipulated the levels of SF2/ASF in HEK293T cells by transfecting cells with siRNA targeting endogenous SF2/ASF, scrambled control siRNA or plasmid to increase levels of SF2/ASF by transient ectopic expression and assayed alternative splicing of endogenous ENSA, SF2/ASF, PABP and NETO2. Fig. 4A shows a Western blot from control cells (lanes 1,2, and 4, respectively) or cells depleted or over expressing SF2/ASF (lanes 3 and 5, respectively). In the context of the ENSA pre-mRNA, the observed binding site is located within an alternative cassette exon. As expected ectopic expression of SF2/ASF increases inclusion of the alternative exon, relative to the cells depleted for SF2/ASF (Fig. 4B). Binding sites for SF2/ASF within the PABP and Neto2 pre-mRNAs are in exons proximal to alternative cassette exons. We found that ectopic expression of SF2/ASF causes increased skipping of the alternative cassette exon in both cases (Fig. 4B). Finally, the observed binding site for SF2/ASF within its' own pre-mRNA (transcribed from the SFRS1 gene) is located near an ultra-conserved element adjacent to a retained intron within the 3′UTR [27], [28]. This intron is believed to attenuate expression of the SFRS1 gene through regulated unproductive splicing and translation (RUST) mechanism [27]. We observe that ectopic expression of SF2/ASF increase splicing of this intron and provides supporting evidence for the RUST hypothesis (Fig. 4B).

**Figure 5:**
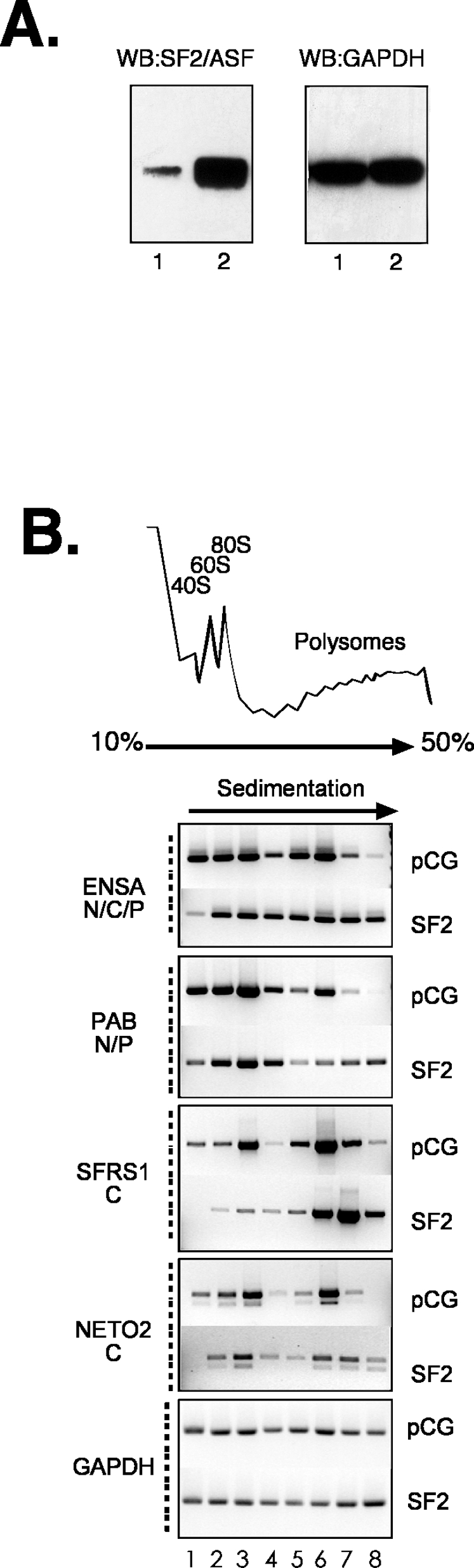
Polyribosome association of endogenous SF2/ASF mRNA targets. A) Western blot analysis of cytoplasmic extracts prepared from cells transfected with pCGT7 of pCGT7 SF2/ASF (lanes 1 and 2, respectively). Blots were probed with anti-SF2/ASF and anti-GAPDH (left and right panel, respectively). B) Polyribosome association of endogenous mRNAs. Cytoplasmic extracts from control or pCGT7 SF2/ASF cells were resolved by sucrose gradient fractionation. The upper panel shows the absorbance of rRNA across the gradient, the positions of ribosomal subunits and complexes are indicated. Lower panels: RT-PCR analysis of RNA targets. Products were resolved by agarose gel electrophoresis. The sub-cellular fraction corresponding to the initial CLIP experiment is indicated below each gene symbol.

The splicing of each of these endogenous pre-mRNA targets is effected by increasing the intracellular concentrations of SF2/ASF. It is known that SF2/ASF can remain associated with mRNA following the splicing reaction and that SF2/ASF can enhance the translation of reporter mRNAs [7], [8]. The CLIP method revealed that a subset of SF2/ASF mRNA targets, including PABP, NETO2, ENSA and SFRS1, were bound by SF2/ASF in the cytoplasm of HEK293T cells. This observation suggests that SF2/ASF may also enhance the translation of these particular mRNA targets. To test this hypothesis we asked whether a specific mRNA target of SF2/ASF exhibited increased association with polyribosomes, the actively translating pool of ribosomes, when SF2/ASF is overexpressed. Cytoplasmic extracts were prepared from HEK293T cells transfected with empty expression vector or epitope tagged SF2/ASF (Fig. 5A). Cytoplasmic extracts were resolved across 10–45% sucrose gradients. Following fractionation, total RNA was extracted from every other fraction and cDNA was synthesized using oligo dT primer. Upon ectopic expression of SF2/ASF all four endogenous mRNA targets showed increased association with polyribosomes (Fig. 5A, lanes 5–8). By contrast, levels of GAPDH remain constant across the gradient when SF2/ASF is overexpressed. We also examined the distribution of several other endogenous targets, but found no difference when SF2/ASF is over expressed, suggesting that this effect is specific for a subset of transcripts rather than a global phenomenon (data not shown). Additionally, there are no obvious differences between the distributions of total RNA across the gradient. These data are in good agreement with our previous findings that SF2/ASF can enhance the polysome association and translation of luciferase reporter mRNAs both *in vitro* and *in vivo* [7]–[9]. Finally these data provide clear evidence that SF2/ASF can drive enhaced polysome association of endogenous mRNAs *in vivo*.

## Discussion

SF2/ASF is a nucleo-cytoplasmic shuttling RNA binding protein with clearly defined activities in spliceosome assembly. Our previous work established a role for SF2/ASF in mRNA translation [7], [8]. These findings suggested that SF2/ASF may co-ordinate the nuclear and cytoplasmic steps of post-transcriptional gene expression for a subset of pre-mRNAs. This hypothesis is further supported by the observation that extrinsic signals leading to activation of the AKT protein kinase enhance the alternative splicing and translation of reporter RNAs containing a consensus SF2/ASF binding site [29]. We used the CLIP protocol to extend our findings to endogenous cellular mRNAs. CLIP allowed us to sample direct mRNA targets of SF2/ASF under conditions that preserve the *in situ* specificity of protein-RNA interactions. By coupling CLIP with sub-cellular fractionation we were able to identify mRNAs associated with SF2/ASF in the nucleus, cytoplasm and in the actively translating pool of ribosomes (Fig. 2 and Table S1). These data revealed a set of mRNAs whose splicing and translation may be coordinated by SF2/ASF. Collectively, our data do not support the view that SF2/ASF plays a general role in mRNA translation, but suggests that the biological importance of this aspect of SF2/ASF function may be at transcript and cell types-specific events. Our recent findings that mTOR is critical to translational control by SF2/ASF suggest that SF2/ASF may connect cellular signal transduction pathways with post-transcriptional control of specific target mRNAs [9].

Using the CLIP approach we were able to not only identify potential RNA targets for SF2/ASF but also define a consensus binding motif present in exonic binding sites (Fig. 3). This purine-rich motif closely resembles the well-characterized exonic splicing enhancers (ESE) present in the fibronectin EDA alternative cassette exon as well as exon 5 of the cardiac troponin T (cTNT) gene [30], [31]. The binding specificity of SR proteins has also been extensively studied using SELEX strategies. Our data are in good agreement from results obtained using only the RRM domains of SF2/ASF for *in vitro* evolution of binding sites [19]. By contrast, functional SELEX experiments, in which bona fide ESEs within reporter constructs are replaced by randomized sequence and *in vitro* splicing is driven by a single recombinant SR protein in S100 complementation assays, yielded different consensus motifs for SF2/ASF [18]. The functional SELEX motifs are more heterogeneous in nature but may represent binding sites for SF2/ASF that can lead to productive splicing. The differences between motifs identified by functional SELEX and CLIP may reflect inherent differences in the experimental conditions. For example, photo cross-linking of protein-RNA interactions is believed to be fairly inefficient, but it allows for recovery of sequences under stringent conditions. It remains possible that sequences identified by CLIP represent only a subset of possible binding sites that are both abundant and suitable for cross-linking. Data presented in Fig. 4B demonstrates that SF2/ASF can regulate the alternative splicing of a subset of pre-mRNAs suggesting that the observed binding sites are functionally relevant. Indeed, 26 out of 44 endogenous pre-mRNAs containing binding sites for SF2/ASF within or proximal to cassette exons appear to be functionally relevant when tested by RT-PCR (Fig. 4B and data not shown). Thus we feel the CLIP method has captured a subset of biologically relevant SF2/ASF binding sites.

Cataloguing the collection of *cis*-acting elements directing post-transcriptional gene regulation on a global scale is only at the initial stages of investigation. *In silico* studies have successfully elucidated *cis*-acting elements that are enriched or co-evolve with constitutive and alternative exons. Several recent computational screens have defined thousands of hexa- or octamers as splicing regulatory sequences [32]–[34]. If a combinatorial code governing splice site selection exists, the solution will likely emerge through interdisciplinary approaches merging computational methods with biochemical and functional genomics approaches. Methods such as SELEX, CLIP and RIP-Chip have the potential to illuminate the specificity and functions of RNA binding proteins by making connections between trans-acting RNA binding proteins and the biological processes they regulate [13], [35]–[37]. CLIP also provides the added value of determining potential mechanisms of RNA binding protein action by revealing the positional context of *cis*-acting RNA elements within a transcript [13]. RNA binding protein target specificity can also be evaluated using platforms to profile alternative splicing such as exon tiling arrays, spliced junction array platforms and RNA-Seq [38]–[40]. Discrimination of direct versus indirect effects is a limitation of these experiments however this may be overcome by applying computational models of consensus binding sites generated by CLIP or SELEX to classify global changes in alternative splicing [39], [41]. Our initial CLIP experiment with SF2/ASF revealed just a small snap shot of putative RNA targets. One clear limitation of this study is the inefficiency of the Sanger sequencing. Merging CLIP with high throughput genome wide sequencing platforms such as Solexa and 454 (Illumina, USA and Roche Diagnostics, USA, respectively), will vastly expand our ability to define the RNA binding specificity. These data will allow for a high resolution map of SF2/ASF binding sites that not only illuminate mechanisms of action in pre-mRNA splicing but also reveal connections to important biological processes such as genome stability, cellular transformation and non-coding RNA processing.

## Materials and Methods

### Cell culture and transient transfection

For CLIP analysis of SF2/ASF, HEK293T cells were grown to 75–80% confluence at 37°C, 5% CO_2_. HEK293T cells were cultured in DMEM containing 10% Fetal Calf Serum and antibiotics. Transient transfection of HEK293T cells with plasmid DNA was performed using Lipofectamine 2000 (Invitrogen) following the manufacturers instructions. Transient transfection of siRNA duplexes and plasmid DNA were performed using Duo-fect transfection reagent (Dharmacon) following the manufacturers specifications. Cells were harvested 48 hours following transfection. siRNA transfection efficiency was monitored using siGLO green siRNA duplexes (Dharmacon) and was found to be 80–95% efficient.

### Plasmids and siRNA

T7 Epitope tagged SF2/ASF was expressed from the pCGT7 vector and has been described previously [42]. siRNA *Smartpools* targeting the endogenous SF2/ASF transcript, or scrambled nontargeting siRNA duplexes were purchased from Dharmacon.

### Cross-linking Immunoprecipitation of SF2/ASF from HEK293T cells

CLIP analysis of SF2/ASF was performed as described by Ule et al.[32] with the following modifications relating to extract preparation and RNase treatment. Nuclear and cytoplasmic extracts were prepared from UV-treated or control cells as previously described [7]. The soluble extract was treated with 30 U RQ DNase 1 for 20 min at 37°C. The reactions were terminated by the addition of 20 mM EDTA. Subsequently, ribosomal subunits were cleared by centrifugation of the extract at 100,000× g using an Optima Max ultracentrifuge (Beckman Coulter, USA) in a TLA120.2 rotor for 20 min. Cleared extracts were then treated with a dilute cocktail of RNase A/T1 (Ambion, USA) at a final dilution range of 1∶1000–1∶10,000 for 20 min at 37°C. 200 U RNaseOut (Invitrogen, USA) was then added to the extract. Proteins were then partially denatured by addition of an equal volume of buffer A (2× PBS, 0.2% SDS, 1% NP-40). An aliquot of each UV-treated extract was used to prepare input RNA fragments. The remainder of the extract was used for immunoprecipitation with anti-SFRS1 monoclonal antibody.

### Characterization of the CLIP tags

All Human exon and gene information was downloaded from Ensembl, using the BioMart tool. The sequence data was downloaded using Perl scripts to access the ensembl database through the Ensembl Core Perl API. The CLIPS sequences were mapped against unspliced gene, cDNA and coding sequences using Mega BLAST (Zhang et al. 2000). The unambiguous mapping of the CLIP tags allowed us to match the CLIPs to Ensembl gene ids. The characterization of type of splicing event that occurs on the alternatively spliced exons that were found to be bound by SF2/ASF was performed according to Ensembl exon information.

### Identification of the SF2/ASF binding site

The distinct CLIP tags that match exons were used on the MEME (Bailey et al., 2006) web server (http://meme.sdsc.edu/meme/meme.html) to identify patterns that are probabilistically significant. The intronic set of CLIP tags was used in the same way but did not result in any meaningful pattern.

### The genome wide distribution of the SF2/ASF binding site pattern

To identify the genome-wide distribution of SF2/ASF binding sites around exon splice sites, for each exon from a protein coding gene (greater than 100 bp), we generated a composite sequence made of the 75 bp upstream of the 3′ splice site, the 5′ exonic 25 bp, the 50 bp from the middle of the exon, the 25 bp 3′ exonic and 75 bp downstream of the 5′ splice site. There were in total 174,440 exons that satisfied this criteria. These were then analyzed using the binding sites in CLIP tags that were identified by MEME.

### Parsing of Data

All parsing of data was performed using multiple Perl scripts.

### *in vivo* splicing assay

HEK293T cells were transfected as described above. Cells were harvested 48 hours post-transfection. One aliquot was used for western blot analysis the other was used to prepare total RNA from the cytosolic fraction. RNA was extracted using TRI Reagent LS (Sigma) following the manufacturers instructions. cDNA was synthesized using oligo dT primer and Superscript RT III (Invitrogen). 50 ng of cDNA was used as a template for RT-PCR analysis. Primers were 5′ end labelled with ^32^PγATP and T4 PNK (NEW England Biolabs). Primers are available upon request. PCR was performed using the following conditions: 3 min at 94°C; 30 cycles of 94°C 30 sec, 59°C 30 sec, 72°C 60 sec; 72°C 5 min. Following PCR, the reactions were ethanol precipitated and resolved upon 10% polyacrylamide/7M urea gels. RT-PCR reactions were visualized by autoradiography or phosphorimager.

### Polyribosome Profiles

Polyribosome profiles were obtained from HEK293T cells as previously described [7], [8]. Total RNA was extracted from each fraction using TRI Reagent LS. Every other fraction was analyzed by RT-PCR. Purification of polyribosome for CLIP analysis of SF2/ASF was accomplished by pelleting polyribosomes through 10–25% sucrose gradients as previously described [8]. Polysome pellets were washed gently with cold PBS, then resuspended in cold 0.5× buffer A (see above). The polysomes were then treated with RQ DNase, and RNase as described in the CLIP protocol [12].

### Supporting Information

A supplementary excel spread sheet accompanies this manuscript (Table S1). It contains complete annotation of the CLIP data.

## Supporting Information

Table S1 A complete list of binding site annotation using the Ensembl, UCSC Known Gene and Rfam databases. The Excel file can be filtered in order to find binding sites identified by CLIP using nuclear, cytoplasmic or polysomal extracts. Headers for the table are as follows: Chromosome: Defines the specific chromosome from the human genome to which the seq-block mapped. Strand: CLIP preserves the orientation of the captured RNA marker, so that it is possible to determine the strandedness of the locus. Id: Generic description given to each binding site during our annotation work flow. Region start/end: The precise chromosomal coordinates defining the unique or overlapping sequence blocks. Regions are define by at least 2 partially overlapping binding sites mapping to the locus. # of fragments in the Cytoplasm/Nucleus/Polysome indicates if the binding site was identified in each compartment. The number specifies whether a sequence block was absent, present in a single assay or multiple assays. # of sample targets is useful for finding binding sites that are in 1, 2 or all three cellular fractions. Gene Annotation: This column describes the relationship of the seq block to annotated protein coding genes based on the UCSC Known Gene Database. Exon Style: This column describes the relationship of the seq block to annotated parts of protein coding genes (exon, intron etc). The strategy is presented in Supplementary Figure 2. USCS Known Gene database ID: This column refers to the name of a specific gene cluster by the UCSC Known Gene database. Gene Symbol: This column contains information pertaining to the approved HUGO Gene Nomenclature Committee symbol for each protein coding gene. Exon Position: This column describes the position of the exon within the protein coding gene. First/Last exon columns: Designation of “1” in either column indicates 5′ or 3′ terminal exon. “1” in both columns denotes that the sequence block is in a single exon gene. Upstream/Downstream Exon Position: These columns are useful for determining the position of introns within a protein coding gene. ncRNA Annotation: Describes the relationship of a sequence block to annotated non coding RNA (ncRNA). Annotation is based on the Rfam database. ncRNA Name: This column describes the gene symbol for each ncRNA containing a sequence block. UTR type: Describes the relationship between sequence blocks and untranslated regions of protein coding genes. Splicing Event: Gives alternative splicing annotation for exonic binding sites based upon AceVIEW, ALT Events and Fast-db, databases.
(0.13 MB XLS)
